# Abstract of Proceedings of the Buffalo Medical Association, July, 7, 1874

**Published:** 1874-08

**Authors:** E. R. Barnes

**Affiliations:** Secretary


ART. III.—*Abstract of the Proceedings of the Buffalo Medical Association.—Tuesday evening July* 7, 1874. Reported by E. R. Barnes, M. D., Secretary.

Members present: The President, Dr. White, in the Chair, and Drs. Rochester, J. F. Miner, Fowler, Samo, W. W. Miner and Barnes.

On motion the reading of the minutes of the last meeting was dispensed with.

Dr. Rochester said there was a good deal of excitement at present in New York city on the subject of Hydrophobia. This excitement had extended to other localities, and many of our own citizens were not free from nervousness on this subject. He had that day been called upon by a lady whose daughter had been bitten by a dog the day before. The animal presented no symptoms of a departnre from health, but the lady had seen a paragraph in one of the New York papers, giving the singular opinion announced by Dr. Hammond, and was greatly alarmed in consequence. The subject of hydrophobia was always interesting, and it had now become prominent. While Dr. Rochester believed that every possible precaution should be taken, by cauterization with a point of nitrate of silver, or by excision, whenever a person is bitten by a dog, whether rabid or free from any symptom of disease, he could not but consider that the enunciation of the doctrine by Dr. Hammond, that the bite of a healthy dog could produce hydrophobia, was deplorable. It would cause much unnecessary alarm, and was founded on insufficient evidence. It cannot be known, frequently, in a case of hydrophobia, whether the animal inflicting the bite was well or not at the time. The symptoms may have been obscure, or when the investigation is made the animal may have recovered. Physicians should receive such a statement as that of Dr. Hammond with the greatest caution. Dr. R. did not believe there was anything to establish such an hypothesis. A man died, it is supposed, of hydrophobia. Certain evidences of disease were found affecting the medulla oblongata. The dog which inflicted the bite was found to be well. Upon this single case, upon so slender an array of facts, the Dr. had made his broad assertion.

It is well, as stated above, to take all possible precaution to prevent injurious effects, whenever a person may be bitten, and it is also important to allay unnecessary fear. Do not kill the dog as is commonly done, but keep him safely and feed him well, and if, at the expiration of one, or at most two weeks, the dog is still free from symptoms of disease, it may be confidently asserted that there is no danger of hydrophobia ensuing as the result of the bite.

The doctrine of Dr. Hammond is likely to do more harm than the superstition long prevalent, that if a person be bitten by a dog, and the animal at any subsequent period should become rabid, the person bitten would then have hydrophobia, which gave rise to endless fears and to prevent which catastrophe the dog was, if possible, at once killed.

It may be said even, that but a small proportion of those who may be bitten by a dog suffering from hydrophobia, contract that disease. Youatt, a reliable authority, states that he has been bitten six times by a dog which he knew to be mad, that he carefully washed the wound and cauterized it with nitrate of silver, and no ill effects ensued.

Dr. Miner said that during the last twenty-five years he had been very frequently called upon by persons who had been bitten. His custom had been to inquire carefully whether there was any reason to think the dog inflicting the injury was not well. Whenever he was satisfied that the dog was well, he had dismissed the patient with the assurance that there was no more danger to be apprehended than if the injury had been inflicted by a nail, a piece of wood, or any blunt instrument. When the person is bitten by a really rabid dog, he would employ excision, or cauterization, or both ; but if even so brief an interval as, say twenty minutes, should elapse before the employment of these measures, it was questionable whether they could be relied upon to prevent infection, any more than they could in the case of a rattlesnake bite, in which we know that absorption of the poison takes place with astonishing rapidity.

Dr. Hammond's one case is of no value as establishing the doctrine to which reference has been made. Repeated experiments and observations would be needed to prove it. He did not know that hydrophobia had ever followed the bite of a healthy dog. Such an assertion is contrary to experience. During twenty-five years, in his own practice, he had never known such an instance. Tetanus might result. As to excising the wound, or borrowing trouble, from the bite of a healthy dog, his experience went to show that both were needless.

Dr. Rochester said he had an idea, not original with himself, that in the case of the bite of a rabid dog, the virus is not absorbed as rapidly as that from the rattlesnake and some insects. The virus of the dog, when introduced into the tissues, lies a certain time dormant. Hydrophobia has not appeared, he believed, in a less time than eight days after the receipt of the bite. From that time up to three weeks, is the common interval ; the average interval being, perhaps, twelve days. Now Dr. Rochester thought that the poison was not at once absorbed. Perhaps the globules were so large that a process of disintegration had to take place, or some other transformation had to be first undergone before the virus could enter the circulation. There were two periods for the use of preventive measures : The first as soon as possible after the bite ; the second, when the wound is cicatrizing, at a time when it takes on a peculiar condition—when it looks blue, is painful and a little elevated. Then the site of the wound may be again excised. The Dr. did not know positively whether this second operation was serviceable or not, but would advise it. He narrated the case of Dr. J. S. Hawley who was called to see a lady who had been bitten by a cat on the bare hand. Whilst searching for the animal Dr. H. was also bitten, through a glove. The lady declined to submit to the measures proposed, but the wound of Dr. Hawley was promptly excised by Dr. White and the cicatrix, on assuming the appearance above described, was also cut out. The woman died, as is supposed, in consequence of the bite, while Dr. Hawley experienced no subsequent ill effects.

Dr. Samo asked how long after the bite, was the cicatrix excised.

Dr. White said, a little over three weeks. In this connection he would refer to a case reported by him about thirty years ago, in Vol. I, Buffalo Medical and Surgical Journal. It was that of a man whom he had previously treated for intermittent fever, and he at first thought that the symptoms presented were those of ague, but it was soon apparent that he had hydrophobia. It was ascertained that he, with two companions, had been bitten by a dog which acted strangely, but which they had not suspected of being rabid. After an incubation of about forty days, hydrophobia was developed and the man died in four or five days. His companions now becoming alarmed, Dr. White excised the cicatrices of their wounds, which had assumed the bluish leaden tint &c., described by Dr. Rochester. They escaped infection. In the case of Dr. Hawley, whose wound he had excised, the hand was protected by a glove, but it was much torn, and the saliva came in contact with the hand. The cicatrix underwent changes, becoming more or less painful, and Dr. White excised this after the death of the woman. After alluding to a number of cases he had seen Dr. W. said: "Whenever a man is bitten, never treat the case lightly—always cauterize or excise. For firstly, if it is a mere punctured wound it will heal more quickly, with less likelihood of tetanus; and secondly, because, while waiting for evidence which may not be attainable, the virus may be absorbed. As to time, use these means instantly if possible, but if a man were bitten by a mad dog ten days ago, or even longer, excision should be practised. Dr. White thought that the poison was introduced somewhat as is the virus in vaccination, which, he believed, could be prevented from producing constitutional effects by excising the point of insertion within four or five days. Certain poisons are not directly absorbed but must first undergo change. Hence excision should be always made, although some time may have elapsed. Dr. White would also recommend re-excision as above described if the dog were rabid. He did not believe with Hammond that the bite of a healthy dog would produce hydrophobia, but he did believe that the poison of a mad dog was absorbed into the blood and acted on the nervous system. Hydrophobia was not tetanus. The composure of mind which resulted to the patient from an assurance of safety was beneficial.

Dr. Samo said that it was desirable to have a better knowledge of the disease of the dog, and cited a case to illustrate the difficulty sometimes of determining whether the dog were rabid or not, especially in the early stages of the disease.

Dr. Miner said that he wished briefly to defend his position. He had no objection to any amount of excision or cauterization if it would prevent all after effects, but to employ them in an ordinary bite was to make a great matter out of nothing. People among the German population were very frequently bitten. To cut out every time and produce alarm by a measure which, even if the dog were mad, is of very doubtful efficacy, was unjustifiable. As to cutting out an inoculation of vaccine virus, can it be done and have all effects of the virus cease? He did not know, but doubted it. As to the globules being too large, or having to undergo a change before absorption, it was uncertain at least. Why is there a period of incubation in Syphilis say of twenty-four days, in vaccination of five days, and in hydrophobia up to one or more years? The explanation presented was very questionable.

Dr. Rochester wished to be put on record as favoring the measures he had advocated. He had but once excised.

Dr. White said the wound would heal more quickly if cauterized and the patients fears be allayed.

Dr. Barnes asked if any case of hydrophobia had been reported.

Dr. Rochester said he thought not. Dogs seemed more irritable though from the heat.

Dr. Barnes presented plaster casts from the lower extremities of an infant about two years and a half old, which had congenital talipes varus effecting the right foot. It will be seen, he said, that the tibia and fibula of the right leg are more than an inch shorter than those of the other extremity. This is a rare complication of talipes, though not extraordinary. Contrary to the appearance usually presented, the internal maleolus of the right leg is very prominent. This would seem to be due to intra-uterine pressure bending the end of the tibia while yet easily moulded. In other respects the deformed limb does not differ materially from the cases ordinarily observed. The left leg is of normal length and the foot is straight; but on the inner aspect of the tibia, at a distance from the head of the tibia, corresponding to the distance of the malleolus of the right leg from the head of its tibia, is a prominence closely resembling on its anterior surface and in its inward curvature, the right internal malleolus. Posteriorly this prominence is deficient in fullness, so that when it is grasped between the thumb and fore fingers, it is felt to be much thinner from before backwards than the right malleolus. By pressing on the parts below the prominence, the tibia can be felt extending to the ankle joint, of normal length, but much smaller in diameter than is natural. Its extremity is small and no indication of a malleolus can be felt at this point. The fibula and external malleolus are well developed and normal. These points can be but imperfectly demonstrated by the casts, representing as they do, only the surface conformation.

It would seem as though the prominence on the left tibia represented an attempt to form here an internal malleolus. It is difficult to explain this formation. The first thought which presented itself was that in early fœtal life the left foot was much inverted by uterine pressure and that a malleolus was actually developed, but that the foot resuming its normal relation to the leg, the intervening space was filled by the imperfect structure which has been pointed out. But it is not easy to reconcile this with the perfection of the fibula which exists, and such a theory may not conform to the facts of intra-uterine growth. Again, the shadowy explanation presented itself of the tendency to symmetrical formation which exists in nature. This would require us to suppose that the deficient length of the right tibia and fibula was an original mal-formation and not due to pressure—a point not without interest in considering the etiology of club foot. Lastly if we suppose that the prominence is caused by the pressure of some portion of the right foot, it is singular that it should so conform in its location and in its anterior and lateral shape to the right malleolus, and it is hard to explain the acute apex, the sharp edge, and the abrupt almost concave posterior surface. Moreover there is no malleolus at the extremity of the bone. The case is presented as having some bearing on the question of the cause of club foot.

Dr. Miner said there was no doubt that most cases of congenital club foot were caused by intra-uterine pressure. Dr. Gross affirms that the etiology is unknown ; but the cause can be demonstrated. Dr. Gross says that pressure would deform other members, and dismissing this as a cause, as well as the theory of an arrest of development, he substitutes the theory of deficient nervous supply, which Dr. M. did not consider an apology for an explanation. Dr. M. had seen cases illustrating arrest of development, such as absence of fingers and toes, but in club foot there is no arrest of development. The parts are natural. There may be diminution in size from atrophy but this is not due to deficient innervation. The amniotic fluid allows free movement of the limbs, and when it is absent or very deficient in quantity, the restraint imposed by the uterine walls may give rise to atrophy. He begged to present a specimen which showed that in this case there was no amniotic fluid whatever in the uterine cavity. Obviously there was none, for the round surface of the fœtus could not be injured except when an arm or a leg was pressed forcibly against it. It is a fœtus of about the fourth month. The whole surface shows evidence of pressure. The right arm is flexed so that the hand is spread out over the left eye, the fore-arm traversing the nose and mouth. The mouth is deformed, the nose flattened, the eye is depressed and the lids tightly closed, and grown together. The left arm crosses the chest, the fingers clasping the right fore-arm, while the pressure from the elbow is such that the hand is bent backwards at the wrist at an acute angle. The right thigh crosses the abdomen, the foot being pressed against the left side. The left lower extremity is turned upwards, and its foot is flattened against the left side of the head. He had other specimens showing the effects of pressure.

Dr. White said that no single cause would explain all cases There were many causes, among them intra-uterine pressure. About thirty-four years ago, in operating for club foot, to which he then paid special attention, he had met a case of double varus in which one limb was four inches shorter than the other.

Dr. Miner said the point of practical importance in the consideration of this subject was the treatment. The best time is as soon as possible after birth. If attended to immediately, the parts could be easily moulded into shape, and retained by adhesive plaster or plaster of paris. Three days after birth was not so good as three hours, such was the rapid change in muscular action. There was not much feeling at that time. Generally it was not necessary to operate when treatment is commenced thus early. At three years of age, the result is never perfect, notwithstanding assertion to the contrary.

Dr. White wished to endorse this statement. At eleven or twelve years of age he thought operation was of little if any use.

Applications for membership of Drs. Hopkins, Chace, W. W. Miner, and Brush were received.

Scarlet fever was reported to be still prevalent.

On motion the society adjourned.

